# Cerebral Metabolism Related to Cognitive Impairments in Multiple System Atrophy

**DOI:** 10.3389/fneur.2021.652059

**Published:** 2021-04-01

**Authors:** Cong Shen, Li Chen, Jing-Jie Ge, Jia-Ying Lu, Qi-Si Chen, Shu-Jin He, Xin-Yi Li, Jue Zhao, Yi-Min Sun, Ping Wu, Jian-Jun Wu, Feng-Tao Liu, Jian Wang

**Affiliations:** ^1^Department of Neurology and National Clinical Research Center for Aging and Medicine, Huashan Hospital, Fudan University, Shanghai, China; ^2^Department of Ultrasound, Huashan Hospital, Fudan University, Shanghai, China; ^3^Positron emission tomography (PET) Center at Huashan Hospital, Institute of Functional and Molecular Medical Imaging, Human Phenome Institute, Fudan University, Shanghai, China

**Keywords:** multiple system atrophy, cognitive impairment, 18 F-fluorodeoxyglucose, positron emission tomography, cerebral metabolism

## Abstract

**Objective:** We aimed to characterize the cognitive profiles in multiple system atrophy (MSA) and explore the cerebral metabolism related to the cognitive decline in MSA using ^18^F-fluorodeoxyglucose (^18^F-FDG) Positron Emission Tomography (PET).

**Methods:** In this study, 105 MSA patients were included for cognitive assessment and 84 of them were enrolled for ^18^F-FDG PET analysis. The comprehensive neuropsychological tests covered five main domains including execution, attention, memory, language, and visuospatial function. The cognitive statuses were classified to MSA with normal cognition (MSA-NC) and MSA with cognitive impairment (MSA-CI), including dementia (MSA-D), and mild cognitive impairment (MSA-MCI). With ^18^F-FDG PET imaging, the cerebral metabolism differences among different cognitive statuses were analyzed using statistical parametric mapping and *post-hoc* analysis.

**Results:** Among 84 MSA patients, 52 patients were found with MSA-CI, including 36 patients as MSA-MCI and 16 patients as MSA-D. In detail, the cognitive impairments were observed in all the five domains, primarily in attention, executive function and memory. In ^18^F-FDG PET imaging, MSA-D and MSA-MCI patients exhibited hypometabolism in left middle and superior frontal lobe compared with MSA-NC (*p* < 0.001). The normalized regional cerebral metabolic rate of glucose (rCMRglc) in left middle frontal lobe showed relative accuracy in discriminating MSA-CI and MSA-NC [areas under the curve (AUC) = 0.750; 95%CI = 0.6391–0.8609].

**Conclusions:** Cognitive impairments were not rare in MSA, and the hypometabolism in frontal lobe may contribute to such impairments.

## Introduction

Multiple system atrophy (MSA) is a sporadic, adult-onset, progressive neurodegenerative disorder, clinically presenting with the combination of parkinsonism, cerebellar ataxia, autonomic failure, and corticospinal disorders (1, 2). Cognitive impairments or dementia are traditionally believed to be rare or even the non-supporting feature in MSA diagnosis (3). Recently, increasing evidences suggested the existence of cognitive decline even dementia in MSA, which could reach up to 10–20% in several studies (4–9). However, the data on the prevalence or the status of cognitive impairments in MSA is greatly limited, which may be partially due to the lack of diagnostic criteria for cognition in MSA (10). In clinical practice, the cognition evaluation in MSA can refer to the clinical diagnostic criteria for mild cognitive impairment and dementia in Parkinson's disease (PD-MCI, PD-D) (11, 12).

As the evidences for cognitive impairments are accumulating, the biomarker and mechanism involved are to be further explored. The degeneration of striatonigral and olivopontocerebellar regions with α-synuclein-immunoreactive inclusions in oligodendrocytes has been acknowledged as the pathological characteristics of MSA (13, 14). In some patients of autopsy-confirmed MSA, neuronal loss in the frontal cortex (15), the α-synuclein pathology in limbic regions or medial temporal lobe (16, 17) were reported to correlate with the cognitive impairments in MSA. Nevertheless, pathological findings in autopsy only represent the end stage of the disease, and the *in vivo* neuroimaging methods may point out more underlying mechanism for further understanding. Currently, most of the neuroimaging studies uncovering cognitive impairments in MSA focused on structural imaging (18–21), while few studies pay attention to ^18^F-fluorodeoxyglucose (^18^F-FDG) positron emission tomography (PET) functional neuroimaging (8, 22–24).

In the current study, we aimed to investigate the detailed cognitive profiles in a Chinese cohort of MSA patients, detect the regional cerebral metabolism differences among different cognitive statuses, and explore the utility of cerebral metabolism in differentiating the cognitive status in MSA.

## Materials and Methods

### Subjects

In this study, 105 patients diagnosed as probable or possible multiple system atrophy (3) were enrolled in Huashan Hospital between February 2012 and August 2020. The diagnosis were made by two senior investigators of movement disorders with face-to-face clinical evaluations. The study was approved by the Human Studies Institutional Review Board, Huashan Hospital, Fudan University. All participants provided written informed consent in accordance with the Declaration of Helsinki before entering this study.

### Clinical Assessments

We systemically collected the demographic information of the patients, including the age, sex, disease duration, and education degree. The motor dysfunction was assessed using Hoehn and Yahr scale and the Unified Parkinson's Disease Rating Scale Part III (UPDRS-III) in the “OFF” state, off anti-parkinsonian medications for at least 12 h.

The cognitive assessments were performed by a comprehensive battery of neuropsychological tests. The global cognitive status was tested through Mini Mental State Examination (MMSE); executive function was tested through the Stroop Color-Word Test (CWT) (25) and Trail-Making Test part B (TMT-B) (26); attention was tested through the Symbol Digit Modalities Test (SDMT) (27) and Trail-Making Test part A (TMT-A) (26); memory function was tested through the Auditory Verbal Learning Test (AVLT) (28) and delayed recall task of the Rey–Osterrieth Complex Figure Test (CFT-delayed recall) (29); visuospatial function was tested through the Clock Drawing Test (CDT) and copy task of Rey–Osterrieth Complex Figure Test (CFT) (29); language ability was tested through Boston Naming Test (BNT) and Animal Verbal Fluency Test (AVFT) (30).

The norm data we used was derived from healthy controls in Shanghai area (31), in which, groups were stratified based on age and educational level. The impairment in each test was defined to be 1.5 SD below mean of the corresponding group in the norm data. The diagnosis of mild cognitive impairment (MCI) and dementia in MSA (MSA-D) were made referring to the standard diagnostic criteria of PD-MCI and PDD (11, 12). The patients who didn't met the criteria of MSA patients with cognitive impairment (MSA-CI) (i.e., deficits present in at least two tests, either within one single cognitive domain or across different domains), including MSA-D and MSA-MCI, were considered as MSA with normal cognition (MSA-NC).

### PET Imaging and Data Analysis

All patients underwent ^18^F-FDG PET imaging within 1 month around clinical assessment. But among 105 patients, 21 patients' imaging were not included for the final PET imaging analysis because of the change of PET/CT machine due to technical issues after May 2019 (Supplementary Figure 1). The subjects were asked to fast for at least 6 h but had free access to water, and anti-parkinsonian medications in patients were withheld for at least 12 h before PET imaging. We used a Siemens Biograph 64 PET/CT (Munich, Germany). Following the CT scan, a PET scan of 10-min duration was started 45-min post-injection. All studies in patients were performed in a resting state in a quiet and dimly lit room (32).

In the stage of data processing, statistical parametric mapping (SPM5) software running in Matlab platform (Mathworks Inc, Sherborn, MA) was applied as described previously (32). First, the original images were spatially normalized into a standard stereotactic Montreal Neurological Institute (MNI) space and estimated using default [^15^O]-H2O PET template (www.fil.ion.ucl.ac.uk/spm/spm99.html) (33). Then they were smoothed with a three-dimensional Gaussian filter of 10 mm Full Width at Half Maximum (FWHM) to improve signal to noise ratio.

To confirm and characterize the metabolism pattern of MSA, two-sample *t*-test was used to compare MSA patients (including three separate groups) and healthy controls (*n* = 15) (mean age 58.53 ± 5.74, male: female 7:8, years of education 9.80 ± 4.34) with age as a covariate according to the general linear model at each voxel in SPM. To explore metabolism characteristics of cognitive impairments in MSA, a pairwise comparison was made between MSA-D, MSA-MCI, and MSA-NC with education entered as a covariate. Clusters we reported met certain criteria as follows: significant with peak threshold at *p* = 0.001(uncorrected) over whole brain regions and extent threshold three times over the average cluster size calculated by the model. For a stricter criterion, clusters that survived a False Discovery Rate (FDR) correction at *P* < 0.05 were also explored. Then we constructed the 4 mm-radius spherical volume of interest (VOI) with the circle center at the peak voxel of cluster, and quantified metabolic values in each VOI using ScAnVP software (Version 5.9.1; Center for Neuroscience, the Feinstein Institute for Medical Research, Manhasset, NY). Due to the individual differences in global metabolic value, the normalized regional cerebral metabolic rate of glucose (rCMRglc) was expressed as: [VOI value/whole-brain metabolism] × 50 × 100%.

Exact coordinates and anatomical locations of significant regions were determined by Talairach-Daemon software (Research Imaging Center, University of Texas Health Science Center, San Antonio, TX, USA). In stereotaxic space, the SPM maps for hypermetabolism and hypometabolism were overlaid on a standard T1-weighted magnetic resonance imaging (MRI) brain template.

### Statistical Analysis

The continuous, normally distributed data (including rCMRglc) among the MSA-NC, MSA-MCI, and MSA-D groups were analyzed using one-way analysis of variance (ANOVA) and *post-hoc* Bonferroni's comparison. Non-normally distributed data were analyzed with Mann–Whitney *U*-tests. The categorical variables were compared using Pearson's chi-square test. The accuracy of normalized rCMRglc in constructed VOI to diagnose the cognitive status was tested by receiver operating characteristic curve (ROC) analysis. Correlations between normalized rCMRglc in each significant cluster and test score/time in specific cognitive domain were assessed by Pearson correlation. All the analyses were conducted using the SPSS software (SPSS for Windows, version 22.0; SPSS Inc., Chicago, IL, USA). *P* < 0.05 was considered to be statistically significant.

## Results

Eighty-four patients with ^18^F-FDG PET imaging (51 males and 33 females) were finally included for analysis in the current study. The mean age of them was 57.12 ± 8.01 years old with the mean disease duration 26.80 ± 20.69 months. The detailed demographic and clinical characteristics were summarized in Table 1. No significant differences of characteristics were found between the included MSA patients (84 cases) with ^18^F-FDG PET and the total MSA patients (105 cases) (Supplementary Table 1).

**Table 1:** Demographic and clinical characteristics in three cognitive statuses of 84 MSA patients with PET imaging.

	**MSA with PET (*n* = 84)**	**MSA-NC (*n* = 32)**	**MSA-MCI (*n* = 36)**	**MSA-D (*n* = 16)**	***p***
Age (years)	57.12 ± 8.01	58.03 ± 7.49	55.22 ± 9.02	59.56 ± 5.72	0.141
Sex (male/female)	(51/33)	(25/7)	(20/16)	(6/10)	0.018
Education (years)	10.82 ± 3.19	11.91 ± 2.98	10.33 ± 3.55	9.75 ± 2.05	0.039
MSA duration (months)	26.80 ± 20.69	24.03 ± 16.00	25.64 ± 17.52	34.94 ± 32.15	0.208
UPDRS III score_OFF	33.58 ± 15.42	29.97 ± 12.68	33.58 ± 15.69	40.81 ± 18.06	0.070
Hoehn & Yahr score	2.94 ± 0.88	2.72 ± 0.73	2.92 ± 0.99	3.44 ± 0.73	0.022

*Data are shown in mean ± SD or (n/m)*.

*p-value represents the significance level of the analysis of variance performed for each item across the three cognitive statuses of 84 MSA patients*.

*MSA-NC, Multiple system atrophy with normal cognition; MSA-MCI, Multiple system atrophy with mild cognitive impairment; MSA-D, Multiple system atrophy with dementia; UPDRS, Unified Parkinson's Disease Rating Scale*.

### Cognitive Profile in MSA Patients

As shown in Table 2, although only 18 (21%) patients had impaired MMSE in global cognitive function, a large proportion of MSA patients had impairments in the 10 individual neuropsychological tests. Among these tests, SDMT had the highest prevalence for impairment (51%), along with TMT-A (42%) and TMT-B (37%), while the two tests with the lowest prevalence for impairment were AVFT score (25%) and CDT score (25%). Impairments in 5 cognitive domains were all observed in MSA patients. The respective numbers and percentages of impairments in each domain were as follows: attention, 48 (57%); executive function, 45 (54%); memory, 43 (51%); visuospatial function, 34 (40%); language, 31 (37%).

**Table 2:** Comparisons of cognitive impairment in the three cognitive statuses of 84 MSA patients with PET imaging.

	**MSA with PET (*n* = 84)**	**MSA-NC (*n* = 32)**	**MSA-MCI (*n* = 36)**	**MSA-D (*n* = 16)**
**MMSE**	26.43 ± 2.80	28.00 ± 1.72	26.92 ± 1.50	22.19 ± 2.61
	18 (21%)	2 (6%)	0 (0%)	16 (100%)
**Executive function**	45 (54%)	6 (19%)	24 (67%)	15 (94%)
CWT-C score	25 (30%)	6 (19%)	12 (33%)	7 (44%)
TMT-B time	31 (37%)	0 (0%)	18 (50%)	13 (81%)
**Attention**	48 (57%)	4 (13%)	29 (81%)	15 (94%)
TMT-A time	35 (42%)	0 (0%)	22 (61%)	13 (81%)
SDMT score	43 (51%)	4 (13%)	25 (69%)	14 (88%)
**Memory**	43 (51%)	4 (13%)	25 (69%)	14 (88%)
AVLT	29 (35%)	2 (6%)	18 (50%)	9 (56%)
CFT-delay recall	29 (35%)	2 (6%)	17 (47%)	10 (63%)
**Visuospatial function**	34 (40%)	6 (19%)	18 (50%)	10 (63%)
CFT score	27 (32%)	2 (6%)	16 (44%)	9 (56%)
CDT score	21 (25%)	4 (13%)	10 (28%)	7 (44%)
**Language**	31 (37%)	0 (0%)	20 (56%)	11 (69%)
AVFT score	21 (25%)	0 (0%)	13 (36%)	8 (50%)
BNT score	22 (26%)	0 (0%)	14 (39%)	8 (50%)

*Data are shown in mean ± SD or n(m%). n(m%) means number and percentage of impairment in each item*.

*MSA-NC, Multiple system atrophy with normal cognition; MSA-MCI, Multiple system atrophy with mild cognitive impairment; MSA-D, Multiple system atrophy with dementia; MMSE, Mini Mental State Examination; CWT-C, Stroop Color-Word Test C; TMT, Trail Making Test; SDMT, Symbol Digit Modalities Test; AVLT, Auditory Verbal Learning Test; CFT, the Rey–Osterrieth Complex Figure Test; CDT, Clock Drawing Test; AVFT, Animal Verbal Fluency Test; BNT, Boston Naming Test*.

MSA patients were classified into three categories: 32 (38.1%) were MSA-NC, 36 (42.9%) were MSA-MCI and 16 (19.0%) were MSA-D. The percentage of MSA-CI reached 61.9%. In accordance with the criteria of three cognitive statuses, MSA-D patients had the poorest performance in MMSE and nearly every neuropsychological test, followed by MSA-MCI and MSA-NC (Supplementary Table 2). The impairment pattern in cognitive domain was similar across MSA-D and MSA-MCI: prominent in attention and executive dysfunction and less prominent in visuospatial function and language. The information of the cognition in the 105 total patients was listed in Supplementary Table 3, showing an analogous pattern.

### Regional Differences of Cerebral Metabolism Across Three Cognitive Statuses

Compared with 15 healthy controls, metabolic characteristics of MSA patients, MSA-D, MSA-MCI, and MSA-NC were all in accordance with MSA-related pattern (MSARP) observed by previous study (34, 35) (Supplementary Figure 2).

**Figure 1:**
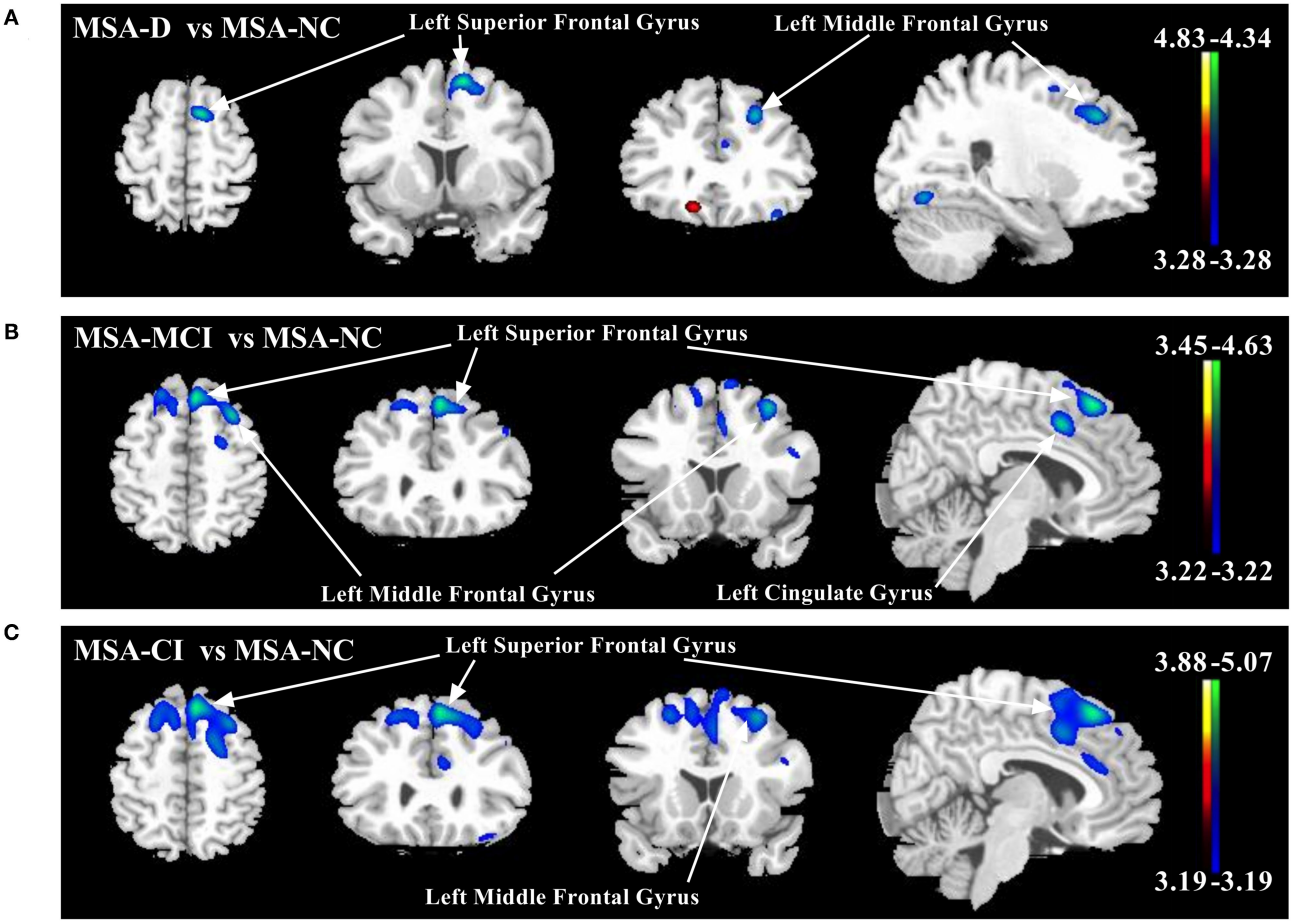
Comparison of regional cerebral metabolic changes across three cognitive statuses (MSA-D, MSA-MCI, and MSA-NC) utilizing voxel-based SPM analysis. **(A)** MSA-D patients displayed hypometabolism (blue–green) in left middle frontal gyrus and superior frontal gyrus compared with MSA-NC. **(B)** MSA-MCI patients displayed hypometabolism in left middle frontal gyrus, bilateral superior frontal gyrus, and cingulate gyrus. **(C)** MSA-CI patients displayed hypometabolism in left middle frontal gyrus and superior frontal gyrus compared with MSA-NC. All changes of metabolism are overlaid on a structural MRI brain template. White arrows indicate the representative brain regions. The thresholds of the color bars depict *T-*values and voxel threshold was set at *p* < 0.001.

Global metabolic values had no significant differences among three cognitive statuses (ANOVA: *F*−0.056, *p* = 0.946). However, several regions with significantly different metabolism by paired-comparison between MSA-D, MSA-MCI, and MSA-NC were detected (Table 3 and Figure 1). Compared with MSA-NC, MSA-D patients revealed hypermetabolism in right cuneus, precentral gyrus, and hypometabolism in left middle frontal gyrus and superior frontal gyrus (*p* < 0.001), similarly, MSA-MCI patients presented less FDG uptake in left middle frontal gyrus, bilateral superior frontal gyrus, and cingulate gyrus (*p* < 0.001). If MSA-CI was regarded as a whole, MSA-CI patients showed the same picture in decreasing metabolism in left middle frontal gyrus and superior frontal gyrus compared with MSA-NC (*p* < 0.001). However, none was found in metabolic differences between MSA-D and MSA-MCI.

**Table 3:** Brain regions with significant metabolic differences between three cognitive statuses of 84 MSA patients.

**Groups**	**Regions**	**BA**	**MNI coordinate**			***Z*max**	**Cluster size (mm^**3**^)**
			***x***	***y***	***z***		
**MSA-D vs. MSA-NC**							
Increased metabolism	Right Cuneus	19	32	−92	32	4.32	1,224
	Right Precentral Gyrus	4	36	−24	42	3.81	864
Decreased metabolism	Left Superior Frontal Gyrus	6	−12	8	60	3.94	1,752
	Left Middle Frontal Gyrus	9	−24	32	38	3.74	1,312
	Left Middle Frontal Gyrus	8	−28	20	48	3.24	1,312
**MSA-MCI vs MSA-NC**							
Increased metabolism	/						
Decreased metabolism	Left Superior Frontal Gyrus	8	−6	28	54	4.29	6,920
	Left Middle Frontal Gyrus	8	−32	16	52	4.05	6,920
	Left Cingulate Gyrus^†^	32	−6	10	42	4.09	6,920
	Right Superior Frontal Gyrus	8	18	32	52	3.64	1,784
	Right Superior Frontal Gyrus	6	10	18	58	3.45	1,784
	Right Cingulate Gyrus	32	10	24	50	3.48	1,784
**MSA-CI vs. MSA-NC**							
Increased metabolism	/						
Decreased metabolism	Left Superior Frontal Gyrus^†^^#^	8	−6	28	54	4.71	18,200
	Left Superior Frontal Gyrus^†^^#^	6	−12	8	64	4.16	18,200
	Left Middle Frontal Gyrus^†^^#^	8	−30	18	50	4.19	18,200

*Statistical threshold: p < 0.001*.

# *survived after FDR correction, p < 0.05*.

† *represents the four representative clusters chosen to construct VOI, make quantification and do post-hoc analysis*.

*BA, Brodmann area; MNI, Montreal Neurological Institute; MSA-D, Multiple system atrophy with dementia; MSA-MCI, Multiple system atrophy with mild cognitive impairment; MSA-NC, Multiple system atrophy with normal cognition; FDR, False Discovery Rate*.

**Figure 2:**
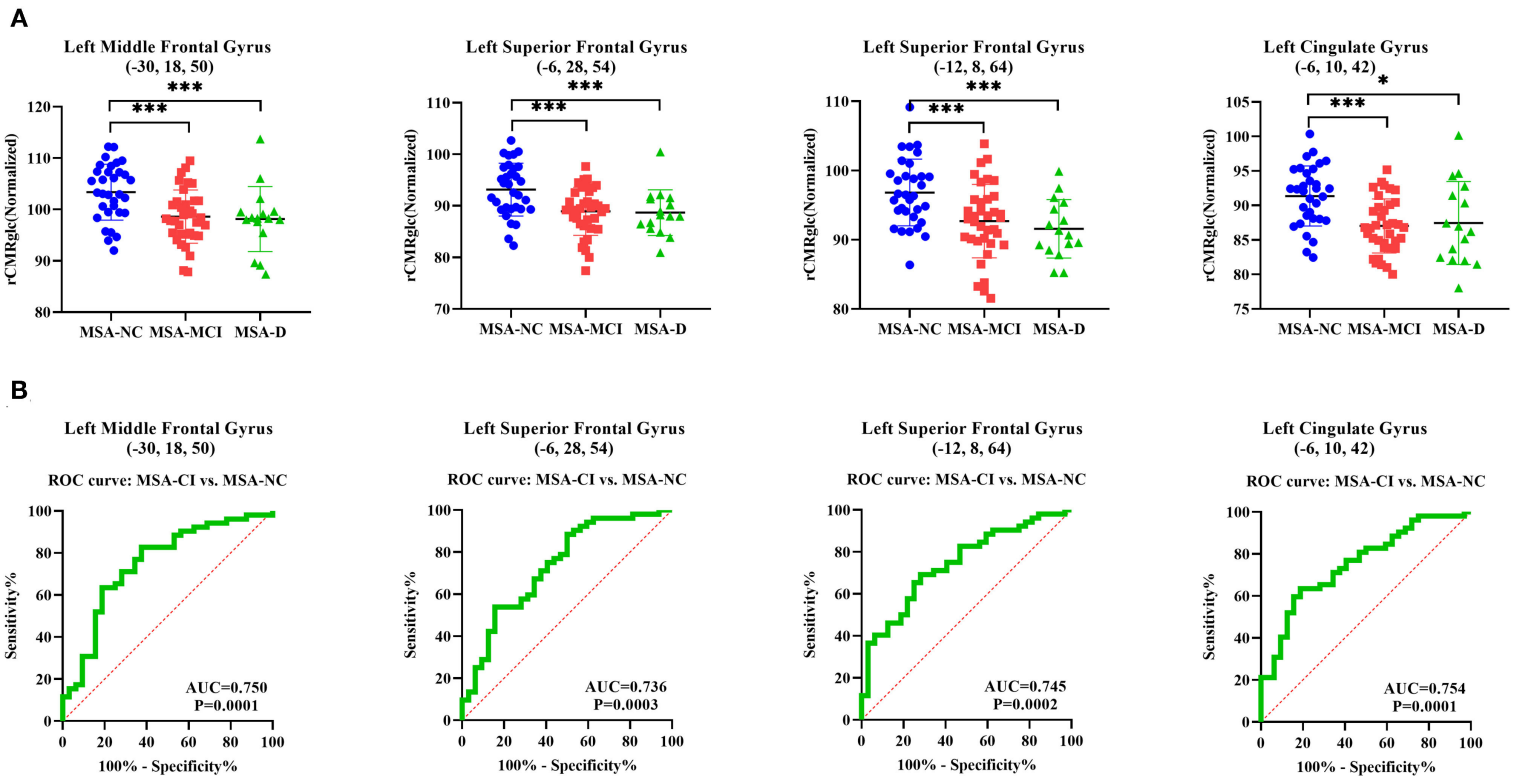
Differences of normalized regional cerebral metabolic rate of glucose (rCMRglc) in representative regions across three cognitive statuses illustrated by *post-hoc* ANOVA and differential diagnostic utility of rCMRglc in corresponding regions for MSA-CI and MSA-NC. **(A)** Normalized rCMRglc obtained within a spherical VOI (4-mm radius) with the center in peak voxel of left middle frontal gyrus cluster (−30, 18, 50), left superior frontal gyrus cluster (−6, 28, 54), left superior frontal gyrus cluster (−12, 8, 64), and left cingulate gyrus cluster (−6, 10, 42) were significantly different among three cognitive statuses. ***p < 0.001; *p < 0.05. **(B)** The area under ROC curve of using rCMRglc in the aforementioned regions to distinguish MSA-CI from MSA-NC were 0.750, 0.736, 0.745, and 0.754, respectively.

Normalized rCMRglc in VOI centered at peak voxel in four representative regions—left middle frontal gyrus cluster (−30, 18, 50), left superior frontal gyrus cluster (−6, 28, 54), left superior frontal gyrus cluster (−12, 8, 64), and left cingulate gyrus (−6, 10, 42) were all significantly different across MSA-D, MSA-MCI, and MSA-NC (ANOVA: *p* < 0.001; Figure 2A). The area under the ROC curve (AUC) of determining diagnostic accuracy of normalized rCMRglc in aforementioned four regions for discriminating between MSA-CI and MSA-NC was moderate at 0.750 (95%CI = 0.6391–0.8609), 0.736 (95%CI = 0.6242–0.8481), 0.745 (95%CI = 0.6387–0.8505) and 0.754 (95%CI = 0.6489–0.8583), respectively (Figure 2B). In addition, AUC using rCMRglc value to distinguish MSA-D and MSA-NC, MSA-MCI and MSA-NC was shown in Supplementary Figure 3.

### Correlation of Test Score in Specific Cognitive Domain With Cerebral Metabolism in MSA Patients

Global metabolic values were not correlated with any cognitive score in five domains (regression analysis: absolute value *r* ≤ 0.179, *p* ≥ 0.104). However, significant correlations between normalized rCMRglc in all the aforementioned four regions and TMT-B time, TMT-A time, SDMT score, CFT-copy score, and AVFT score were observed (Supplementary Figure 4 and Supplementary Table 4).

## Discussion

Four major findings were reported in this ^18^F-FDG PET imaging study on the cognitive deficits in patients with MSA. First, cognitive impairments were not rare in MSA, with the proportion of MSA-CI up to 62%. Second, cognitive impairments in MSA were broad, covering all the five cognitive domains. Third, comparing with patients with MSA-NC, patients with MSA-CI presented decreased metabolism in left middle frontal gyrus and superior frontal gyrus in ^18^F-FDG PET imaging. Fourth, cognitive scores were significantly correlated with metabolic values in frontal lobe and cingulate gyrus. The latter two findings suggested the hypometabolism in frontal lobe was closely associated with cognitive decline in MSA patients. It is of practical significance to recognize that such hypometabolism in frontal gyrus existed even in non-dementia status (MCI). Further longitudinal study is needed to identify the role of frontal hypometabolism in the evolution process of cognitive impairments in MSA.

In our study, 19.0% of the MSA patients were classified as MSA-D, 42.9% patients as MSA-MCI, and 38.1% patients presented with normal cognitive function. Consistent with our report, emerging evidences (4–8, 36) supported the cognitive impairments in MSA. In another Chinese cohort (5), Cao reported that 32.7% MSA patients presented global cognitive deficits based on the Addenbrooke's Cognitive Examination-Revised (ACE-R). In Richard's study from UK (6), nearly 20% MSA patients were considered as MSA with cognitive impairment according to the Mattis Dementia Rating Scale (DRS). One of the main reasons causing the prevalence disparity in cognitive impairments in MSA may be the lack of accurate criteria for the cognitive impairments in MSA and corresponding tests (10). Therefore, the cognition changes in MSA may be underestimated and the development of sensitive criteria are in need.

Moreover, cognitive impairments of MSA patients in our study presented broad neurocognitive phenotypes. Attention (57%), executive function (54%), and memory (51%) were the three domains dominantly influenced. In a previous study using similar neuropsychological tests covering the five domains (8), 65.7% MSA patients had impairment in memory, 48.6% patients in executive dysfunction, supporting our findings. Apart from the impairments in the field of attention, executive function and memory, the impairment proportions in the field of visuospatial function and language were nearly 40% of total. Hence, our results suggested a wide range of deficits in cognitive domains in MSA, and such cognitive impairment feature should be further verified.

The evidences for cognitive impairments in MSA are accumulating, but the biomarkers and mechanisms involved remain to be further elucidated. In the current study with ^18^F-FDG PET imaging, MSA-D and MSA-MCI patients showed decreased metabolism in the frontal lobes comparing with MSA-NC, and the normalized rCMRglc was accurate in discriminating MSA-CI and MSA-NC. Cortical hypometabolism seen in the past FDG studies (Supplementary Table 5) was supportive of our result, but their classification for cognitive statuses was not elaborate as us (23). In previous MRI studies (Supplementary Table 5), patients with MSA-CI showed volume reduction in the frontal, temporal cortical areas (18–20), corpus callosum (21), basal ganglia (19), parahippocampal and lingual cortices (7). Howerver, the hypometabolism in MSA-CI was found mainly in the left frontal gyrus in our ^18^F-FDG PET imaging, suggesting such hypometabolism we reported was not identical to the structure volume reduction, inspiring us to further explore the role of frontal hypometabolism in the cognitive declines of MSA.

We believed that the frontal hypometabolism shared some neuropathological bases (Supplementary Table 5). In patients with MSA-D, microscopically neuronal cell loss, gliosis, and glial cytoplasmic inclusions (GCIs) accumulation were reported (37, 38). In Salvesen's stereological study, fewer frontal cortex neurons were found in patients with executive dysfunction than those with normal executive function (15). In term of burders of GCIs or neuronal cytoplasmic inclusion (NCI) in middle frontal gyrus, however, no difference was found between MSA-CI and MSA-NC (17, 39). Therefore, the detailed mechanisms involved in the cognition-related hypometabolism need to be further explored.

The strengths in our study are as follows. First, we got relative large sample size with systematic neuropsychological tests and ^18^F-FDG PET imaging in MSA patients. Second, in terms of cognitive status in assessment, we defined MSA-MCI as a non-dementia stage referring to PD-MCI and detected existing metabolic changes in MSA-MCI state, which has not been previously reported. However, there are several limitations in our study. First, we used UPDRS-III as a index to assess the movement ability of MSA patients and we admit that Unified Multiple System Atrophy Rating Scale (UMSARS) would be better and more systematic. But unfortunately, we hadn't got the Chinese version of UMSARS at the time of the cohort enrollment. Second, we didn't do partial volume effect (PVE) correction due to the incomplete data in MRI, so we constructed a relatively narrow VOI to quantify metabolic values in order to reduce the impact of atrophy on hypometabolism. We expect to obtain rigorous results based on PVE correction in the future study with newly recruited patients. Third, although neuropsychological tasks and indicators less affected by motor disability were selected in our study and evaluated when the patient was in relatively good condition, cognitive performance was biased by motor disorder. And in the future study, we will develop more suitable tests to compensate for this bias. Fourth, the fewer sample size in MSA-D might cause the smaller cluster sizes of hypometabolism in MSA-D than the ones observed in MSA-MCI. And we hope to perform a study with larger sample size in the future. Fifth, the hypermetabolism in cuneus and precentral gyrus observed in MSA-D compared with MSA-NC was unique, which we speculated as a compensation. But so far there was few evidence on neuropathological burdens or structural changes in these areas. Therefore, future studies are necessary to confirm this result and make explanations for underlying mechanism.

In conclusion, we reported a more frequent and wider range of cognitive impairments in MSA patients than we previously considered. In ^18^F-FDG PET imaging, regional hypometabolism in the left middle and superior frontal gyrus was verified as an characteristic biomarker to the cognitive deficits in MSA. Longitudinal studies are needed to clarify the role of frontal hypometabolism in the development of cognitive impairments in MSA.

## Data Availability Statement

The raw data supporting the conclusions of this article will be made available by the authors, without undue reservation.

## Ethics Statement

The studies involving human participants were reviewed and approved by Human Studies Institutional Review Board, Huashan Hospital, Fudan University. The patients/participants provided their written informed consent to participate in this study.

## Author Contributions

JW and F-TL were involved in the conception and design of the study. CS, LC, Q-SC, S-JH, X-YL, JZ, Y-MS, J-JW, and PW took part in the acquisition, screening, and analysis of data. CS, J-JG, and J-YL were responsible for figures and tables. CS, LC, and F-TL wrote the first draft of the manuscript. F-TL and JW revised the manuscript. All authors contributed to the article and approved the submitted version.

## Conflict of Interest

The authors declare that the research was conducted in the absence of any commercial or financial relationships that could be construed as a potential conflict of interest.
