# Supplementary material for: Cerebral Metabolism Related to Cognitive Impairments in Multiple System Atrophy
Source: Front Neurol. 2021 Apr 1;12:652059. doi: 10.3389/fneur.2021.652059 (PMC8047308; doi:10.3389/fneur.2021.652059)
Supplement: Supplementary file 1 [file Data_Sheet_1.doc]

**Supplementary Table 1.** Demographic and clinical characteristics in 84 MSA patients with PET imaging and 105 total MSA patients

|  | MSA with PET (n=84) | Total MSA (n=105) | p |
| --- | --- | --- | --- |
| Age (years) | 57.12±8.01 | 57.37±8.02 | 0.830 |
| Sex (male/female) | (51/33) | (63/42) | 0.921 |
| Education (years) | 10.82±3.19 | 10.38±3.39 | 0.363 |
| MSA duration (months) | 26.80±20.69 | 25.90±19.48 | 0.761 |
| UPDRS III score_OFF | 33.58±15.42 | 33.55±15.35 | 0.989 |
| Hoehn & Yahr score | 2.94±0.88 | 2.93±0.90 | 0.956 |

Data are shown in mean± SD or (n/m).

p Value represents the significance level of t-test performed for each item between 84 MSA patients with PET and total 105 MSA patients.

Note: UPDRS = Unified Parkinson’s Disease Rating Scale.

**Supplementary Table 2.** Comparisons of raw data in cognitive tests across the three cognitive statuses of 84 MSA patients with PET imaging

|  | MSA with PET (n=84) | MSA-NC (n=32) | MSA-MCI (n=36) | MSA-D (n=16) | p | Post-hoc significance |
| --- | --- | --- | --- | --- | --- | --- |
| **Executive function** |  |  |  |  |  |  |
| CWT-C score | 41.30±6.82 | 43.53±6.37 | 40.22±7.06 | 39.25±6.26 | 0.054 | / |
| TMT-B time | 203.58±80.81 | 149.66±35.16 | 222.19±74.17 | 269.56±95.21 | <0.0001 | [D<N^c^] [M<N^c^] |
| **Attention** |  |  |  |  |  |  |
| TMT-A time | 90.73±46.98 | 60.78±15.31 | 102.81±48.49 | 123.44±53.24 | <0.0001 | [D<N^c^] [M<N^c^] |
| SDMT score | 23.56±13.09 | 32.63±10.95 | 19.19±10.63 | 15.25±11.89 | <0.0001 | [D<N^c^] [M<N^c^] |
| **Memory** |  |  |  |  |  |  |
| AVLT |  |  |  |  |  |  |
| -Short delayed recall | 3.99±2.25 | 5.31±1.86 | 3.42±1.99 | 2.63±2.25 | <0.0001 | [D<N^c^] [M<N^b^] |
| -Long delayed recall | 3.77±2.22 | 5.03±1.84 | 3.22±2.02 | 2.50±2.25 | <0.0001 | [D<N^c^] [M<N^b^] |
| -cued recall | 3.77±2.42 | 5.16±2.10 | 2.89±2.14 | 3.00±2.48 | <0.0001 | [D<N^b^] [M<N^c^] |
| CFT-delay recall | 11.71±6.97 | 15.41±6.35 | 10.83±6.69 | 6.31±4.36 | <0.0001 | [D<N^c^] [M<N^b^] |
| **Visuospatial function** |  |  |  |  |  |  |
| CFT score | 27.74±7.43 | 32.06±4.36 | 26.78±6.45 | 21.25±9.06 | <0.0001 | [D<N^c^] [D<M^a^] [M<N^b^] |
| CDT score | 19.25±7.10 | 22.03±6.23 | 18.44±6.48 | 15.50±8.21 | 0.006 | [D<N^b^] |
| **Language** |  |  |  |  |  |  |
| AVFT score | 13.37±4.44 | 16.22±3.38 | 11.94±4.37 | 10.88±3.48 | <0.0001 | [D<N^c^] [M<N^c^] |
| BNT score | 21.57±4.37 | 24.03±2.81 | 20.81±4.15 | 18.38±4.88 | <0.0001 | [D<N^c^] [M<N^b^] |

Data are shown in mean± SD.

p Value represents the significance level of the analysis of variance performed for each test score/time across the three groups. ^a^ p < 0.05, ^b^ p < 0.01, ^c^ p < 0.001.

Note: MSA-NC = Multiple system atrophy with normal cognition; MSA-MCI = Multiple system atrophy with mild cognitive impairment; MSA-D = Multiple system atrophy with dementia; CWT-C = Stroop Color-Word Test C; TMT = Trail Making Test; SDMT = Symbol Digit Modalities Test; AVLT = Auditory Verbal Learning Test; CFT = the Rey–Osterrieth Complex Figure Test; CDT =Clock Drawing Test; AVFT = Animal Verbal Fluency Test; BNT = Boston Naming Test.

**Supplementary Table 3.** Comparisons of cognitive impairment in the three cognitive statuses of 105 MSA patients

|  | Total MSA (n=105) | MSA-NC (n=39) | MSA-MCI (n=46) | MSA-D (n=20) |  | |
| --- | --- | --- | --- | --- | --- | --- |
| **MMSE** | 26.42±2.71 | 27.95±1.70 | 26.93±1.45 | 22.25±2.36 | |  |
|  | 22(21%) | 2(5%) | 0(0%) | 20(100%) | |  |
| **Executive function** | 54(51%) | 6(15%) | 29(63%) | 19(95%) | |  |
| CWT-C score | 30(29%) | 6(15%) | 14(30%) | 10(50%) | |  |
| TMT-B time | 38(36%) | 0(0%) | 22(48%) | 16(80%) | |  |
| **Attention** | 58(55%) | 4(10%) | 36(78%) | 18(90%) | |  |
| TMT-A time | 42(40%) | 0(0%) | 26(57%) | 16(80%) | |  |
| SDMT score | 52(50%) | 4(10%) | 31(67%) | 17(85%) | |  |
| **Memory** | 54(51%) | 6(15%) | 31(67%) | 17(85%) | |  |
| AVLT | 37(35%) | 4(10%) | 22(48%) | 11(55%) | |  |
| CFT-delay recall | 35(33%) | 2(5%) | 20(43%) | 13(65%) | |  |
| **Visuospatial function** | 42(40%) | 7(18%) | 22(48%) | 13(65%) | |  |
| CFT score | 34(32%) | 3(8%) | 19(41%) | 12(60%) | |  |
| CDT score | 23(22%) | 4(10%) | 11(24%) | 8(40%) | |  |
| **Language** | 37(35%) | 0(0%） | 23(50%) | 14(70%) | |  |
| AVFT score | 25(24%) | 0(0%） | 14(30%) | 11(55%) | |  |
| BNT score | 27(26%) | 0(0%) | 16(35%) | 11(55%) | |  |

Data are shown in mean± SD or n(m%). n(m%) means number and percentage of impairment in each item.

Note: MSA-NC = Multiple system atrophy with normal cognition; MSA-MCI = Multiple system atrophy with mild cognitive impairment; MSA-D = Multiple system atrophy with dementia; MMSE = Mini Mental State Examination; CWT-C = Stroop Color-Word Test C; TMT = Trail Making Test; SDMT = Symbol Digit Modalities Test; AVLT = Auditory Verbal Learning Test; CFT = the Rey–Osterrieth Complex Figure Test; CDT =Clock Drawing Test; AVFT = Animal Verbal Fluency Test; BNT = Boston Naming Test.

**Supplementary Table 4.** Correlation of test score in specific cognitive domain with cerebral metabolism in MSA patients

|  | Left Middle Frontal Gyrus | | Left Superior Frontal Gyrus^1^ | | Left Superior Frontal Gyrus^2^ | | Left Cingulate Gyrus | |
| --- | --- | --- | --- | --- | --- | --- | --- | --- |
|  | r | p value | r | p value | r | p value | r | p value |
| **MMSE** | 0.222 | 0.043^a^ | 0.220 | 0.045^a^ | 0.172 | 0.117 | 0.142 | 0.196 |
| **Executive function** |  |  |  |  |  |  |  |  |
| CWT-C score | 0.081 | 0.462 | 0.035 | 0.751 | 0.030 | 0.785 | 0.068 | 0.539 |
| TMT-B time | -0.372 | <0.001^c^ | -0.370 | 0.001^b^ | -0.333 | 0.002^b^ | -0.364 | 0.001^b^ |
| **Attention** |  |  |  |  |  |  |  |  |
| TMT-A time | -0.363 | 0.001^b^ | -0.367 | 0.001^b^ | -0.241 | 0.027^a^ | -0.321 | 0.003^b^ |
| SDMT score | 0.459 | <0.001^c^ | 0.341 | 0.001^b^ | 0.334 | 0.002^b^ | 0.389 | <0.001^c^ |
| **Memory** |  |  |  |  |  |  |  |  |
| AVLT |  |  |  |  |  |  |  |  |
| -Short delayed recall | 0.227 | 0.037^a^ | 0.150 | 0.174 | 0.091 | 0.410 | 0.226 | 0.039^a^ |
| -Long delayed recall | 0.216 | 0.048^a^ | 0.174 | 0.114 | 0.106 | 0.335 | 0.271 | 0.013^a^ |
| -cued recall | 0.318 | 0.003^b^ | 0.179 | 0.104 | 0.167 | 0.128 | 0.228 | 0.008^b^ |
| CFT-delay recall | 0.232 | 0.034^a^ | 0.209 | 0.056 | 0.293 | 0.007^b^ | 0.316 | 0.003^b^ |
| **Visuospatial function** |  |  |  |  |  |  |  |  |
| CFT score | 0.293 | 0.007^b^ | 0.310 | 0.004^b^ | 0.319 | 0.003^b^ | 0.278 | 0.010^a^ |
| CDT score | 0.096 | 0.385 | -0.126 | 0.252 | -0.014 | 0.898 | 0.057 | 0.607 |
| **Language** |  |  |  |  |  |  |  |  |
| AVFT score | 0.354 | 0.001^b^ | 0.380 | <0.001^c^ | 0.420 | <0.001^c^ | 0.428 | <0.001^c^ |
| BNT score | 0.162 | 0.142 | 0.007 | 0.952 | 0.089 | 0.419 | 0.249 | 0.023^a^ |

r Value represents the Pearson correlation coefficient evaluating correlations between each test score/time and normalized rCMRglc in specific region.

p Value represents the significance level of Pearson correlation between each test score/time and normalized rCMRglc in specific region.

^a^ p<0.05; ^b^ p<0.01; ^c^ p<0.001.

^1^ represents left superior frontal gyrus clusters centered at (-6, 28, 54), ^2^ represents left superior frontal gyrus clusters centered at (-12, 8, 64).

Note: MMSE = Mini Mental State Examination; CWT-C = Stroop Color-Word Test C; TMT = Trail Making Test; SDMT = Symbol Digit Modalities Test; AVLT = Auditory Verbal Learning Test; CFT = the Rey–Osterrieth Complex Figure Test; CDT =Clock Drawing Test; AVFT = Animal Verbal Fluency Test; BNT = Boston Naming Test.

**Supplementary Table 5.** Summarizing studies evaluating cognitive impairment and neural correlats

| PMID | Year | Sample size | Classification | Cognitive test/ record | Methods | Results |
| --- | --- | --- | --- | --- | --- | --- |
| 26464477 | 2015 | 11 | MSA-IEF, MSA-NEF | Limited tests | Neuropathology | MSA-IEF had fewer neurons in the frontal cortex than MSA-NEF |
| 25981961 | 2015 | 35 | / | Limited tests | Neuropathology | CI was associated with Lewy body-like NI in neocortex |
| 26970514 | 2016 | 12 | MSA-D, MSA-ND | Clinical record | Neuropathology | MSA-D had frequent G-NCIs in medial temporal region |
| 27859650 | 2016 | 102 | MSA-CI, MSA-NC | Clinical record | Neuropathology | MSA-CI had a greater burden of NCI in the dentate gyrus than MSA-NC |
| 32385496 | 2020 | 148 | MSA-CI, MSA-NC | Clinical record | Neuropathology | NCI burden in the hippocampus and parahippocampus was associated with the occurrence of memory impairment |
| 19486137 | 2009 | 23 | / | Comprehensive tests | MRI | Memory scores correlated with pre-frontal lobe atrophy |
| 23529023 | 2013 | 15 | MSA-D, MSA-ND | Comprehensive tests | MRI | Cortical thickness was reduced in left parahippocampal and right lingual cortex in MSA-D than MSA-ND |
| 26202063 | 2015 | 15 | / | Comprehensive tests | MRI | CI correlated significantly with thinning in the neocortex, cerebellum and striatum |
| 26234320 | 2016 | 18 | / | Comprehensive tests | MRI | Atrophy in the thalamus, cerebellum and pericalcarine gyrus were correlated with attentional, executive and visuospatial dysfunctions |
| 27778099 | 2017 | 72 | MSA-CI, NSA-NC | MMSE | MRI | MSA-CI showed only focal volume reduction in the left dorsolateral prefrontal cortex compared with MSA-NC |
| 29974207 | 2018 | 30 | MSA-CI, NSA-NC | ACE-R | MRI | FA values in MSA–CI decreased in the corpus callosum compared with MSA-NC |
| 31069705 | 2019 | 32 | / | Limited tests | MRI | Cognitive scores correlated with the FC values between the cerebellum and medial prefrontal/anterior cingulate cortices |
| 31559533 | 2020 | 26 | / | Comprehensive tests | MRI | Cortical thinning in temporal regions correlated with global cognitive status and memory impairment. |
| 18178568 | 2008 | 37 | / | Comprehensive tests | FDG PET | The hypometabolism spreading pattern from frontal to parieto-temporal cortex coincides with the progressive cognitive decline |
| 31621603 | 2020 | 85 | 3 profile | Limited tests | FDG PET | In profile 3 (with CI), hypometabolism was most pronounced in the right posterior cerebellar region in comparison with profile 1 (without CI) |
| 33137618 | 2020 | 88 | MSA-CI, NSA-NC | MMSE | FDG PET | Metabolism in the posterior cingulate was lower in MSA-CI than MSA-NC |
| **Current study** | | 84 | MSA-D, MSA-MCI, MSA-NC | Comprehensive tests | FDG PET | MSA-D and MSA-MCI patients exhibited hypometabolism in left middle and superior frontal lobe compared with MSA-NC |

Note: MSA-IEF = multiple system atrophy with impaired executive function; MSA-NEF = multiple system atrophy with normal executive function; CI= cognitive impairment; NI= neuronal inclusions; G-NCI= globular neuronal cytoplasmic inclusions; MSA-D= multiple system atrophy with dementia; MSA-ND= multiple system atrophy without dementia; MSA-CI= multiple system atrophy with cognitive impairment; MSA-NC= multiple system atrophy with normal cognition; MSA-MCI= multiple system atrophy with mild cognitive impairment; MRI= magnetic resonance imaging; MMSE= Mini Mental State Examination; ACE-R= Addenbrooke’s Cognitive Examination-Revised; FA= fractional anisotropy; FC= functional connectivity.

Figure legends

**Supplementary Figure 1**. Flowchart of the study.


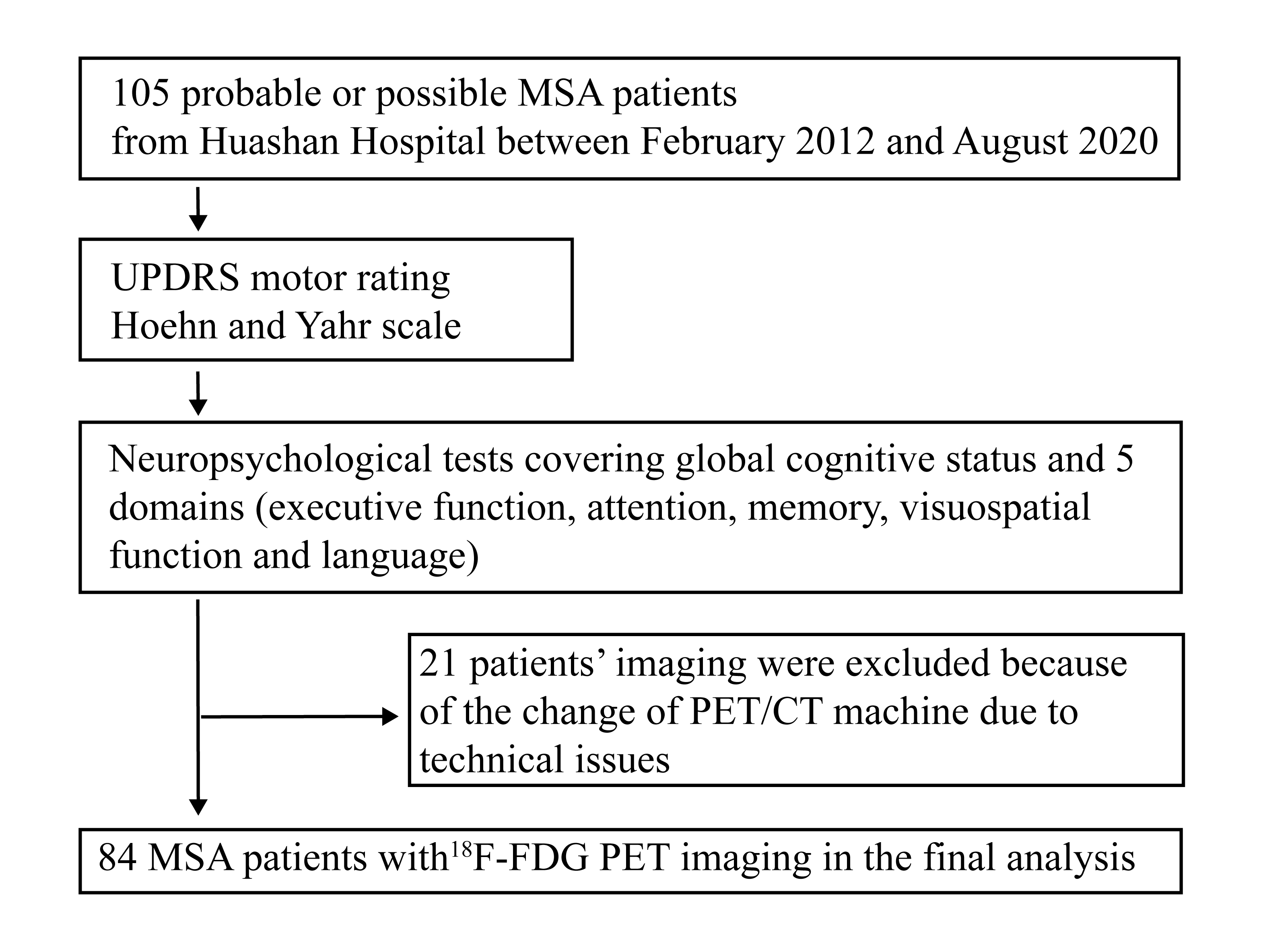


**Supplementary Figure 2.** Comparison of regional cerebral metabolic changes between MSA and HC utilizing voxel-based SPM analysis. **(A)** MSA patients displayed hypometabolism (blue–green) in cerebellum and lentiform nucleus compared with HC. Similar result was in (B-D). **(B)** MSA-D vs HC. **(C)** MSA- MCI vs HC. **(D)** MSA-NC vs HC. All changes of metabolism are overlaid on a structural MRI brain template. White arrows indicate the representative brain regions. The thresholds of the color bars depict T values and voxel threshold was set at p < 0.001.


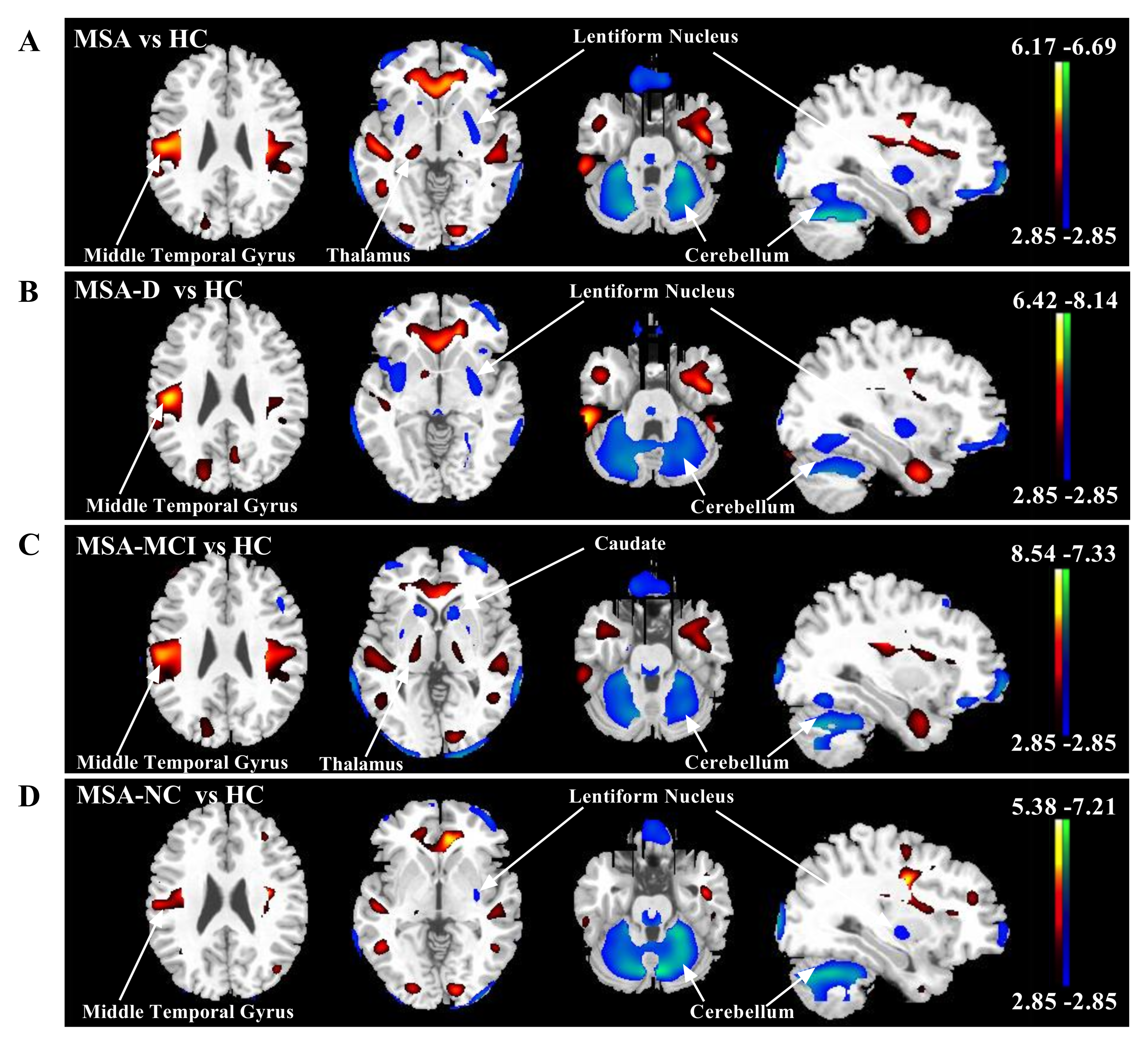


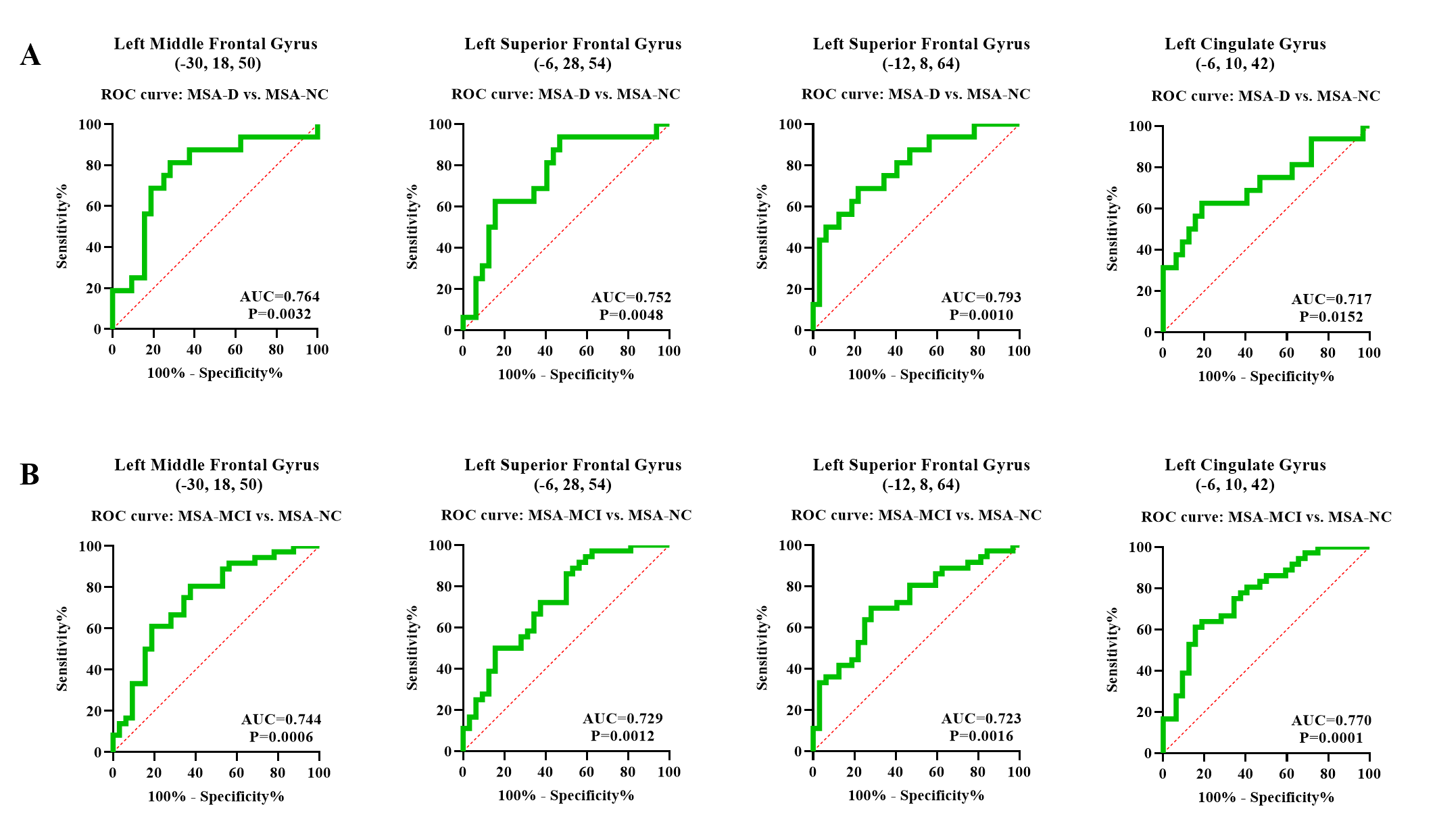


**Supplementary Figure 3.** Differential diagnostic utility of rCMRglc in corresponding regions for MSA-D and MSA-NC, MSA-MCI and MSA-NC. **(A)** The area under ROC curve of using rCMRglc obtained within a spherical VOI (4-mm radius) with the center in peak voxel of left middle frontal gyrus cluster (-30, 18, 50), left superior frontal gyrus cluster (-6, 28, 54), left superior frontal gyrus cluster (-12, 8, 64) and left cingulate gyrus cluster (-6, 10, 42) to distinguish MSA-D from MSA-NC were 0.764, 0.752, 0.793 and 0.717, respectively. **(B)** The area under ROC curve of using rCMRglc in the aforementioned areas to distinguish MSA-MCI from MSA-NC were 0.744, 0.729, 0.723 and 0.770, respectively.


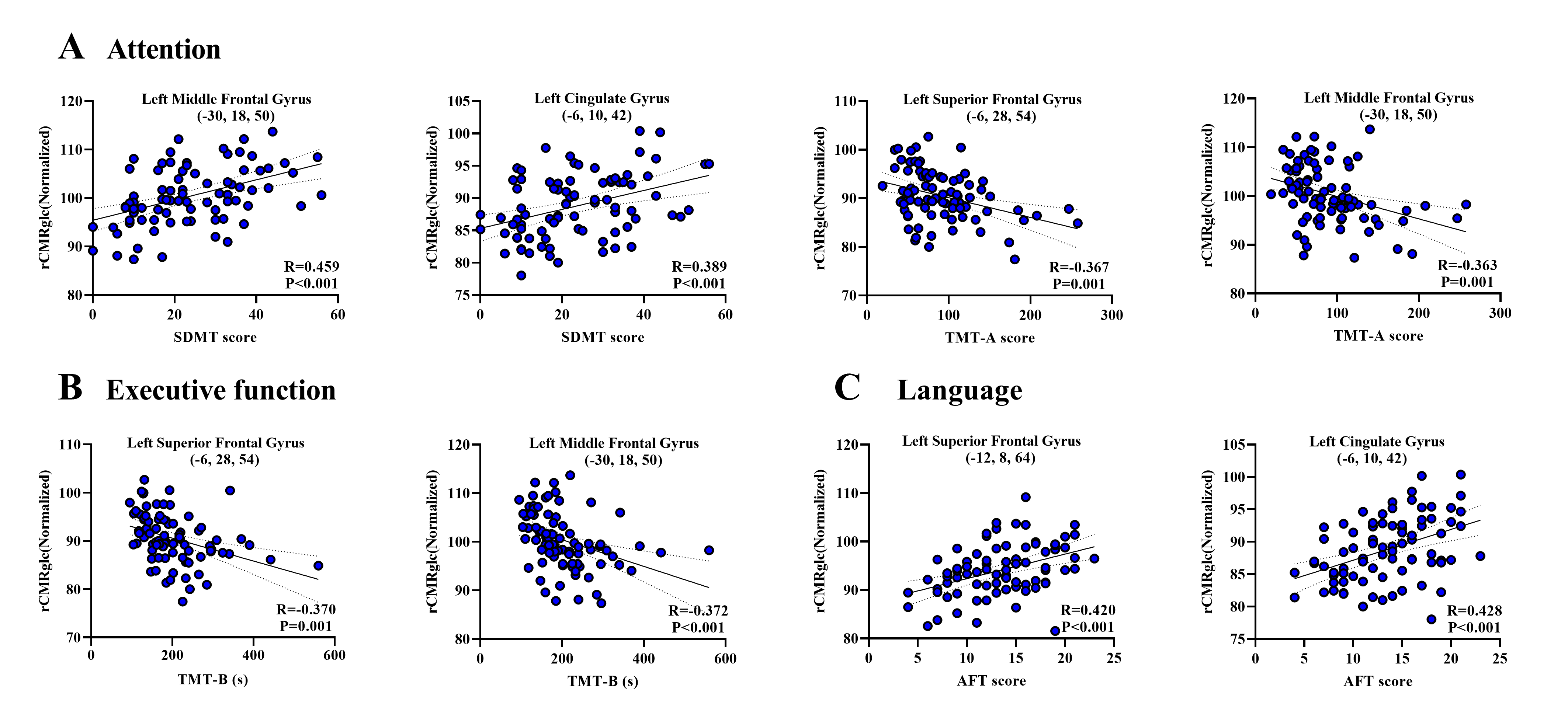


**Supplementary Figure 4.** Scatter plots showing correlations between test score in different cognitive domains and normalized rCMRglc in represntative regions. **(A)** SDMT score in attention domain was positively correlated with normalized rCMRglc in left middle frontal gyrus and left cingulate gyrus. TWT-A time in the same domain was negatively correlated with normalized rCMRglc in left superior frontal gyrus and left middle frontal gyrus. **(B)** TWT-B time in executive function domain was negatively correlated with normalized rCMRglc in left superior frontal gyrus and left middle frontal gyrus. **(C)** AVFT score in language domain was positively correlated with normalized rCMRglc in left superior frontal gyrus and left cingulate gyrus. Each blue-filled circle represents one patient with test score of specific cognitive domain and rCMRglc in certain brain region. R value means the correlation coefficients.
